# Supplementary material for: Predicting visual function by interpreting a neuronal wiring diagram
Source: Nature. 2024 Oct 2;634(8032):113–23. doi: 10.1038/s41586-024-07953-5 (PMC11446822; doi:10.1038/s41586-024-07953-5)
Supplement: Supplementary file 6 — CRF and ERF predictions for individual TmY4 and TmY9 cells. Analogous to Supplementary Data 3, but for TmY target types. Shown are the top four monosynaptic pathways, the strongest pathway passing through each of the top ten intermediary types (ranking from Extended Data Fig. 7), and the trisynaptic pathway Tm1–TmY–Dm3–TmY (see the section entitled Prediction of spatial normalization). [file 41586_2024_7953_MOESM6_ESM.zip › DataS4/TmY9q/720575940631899496.pdf]

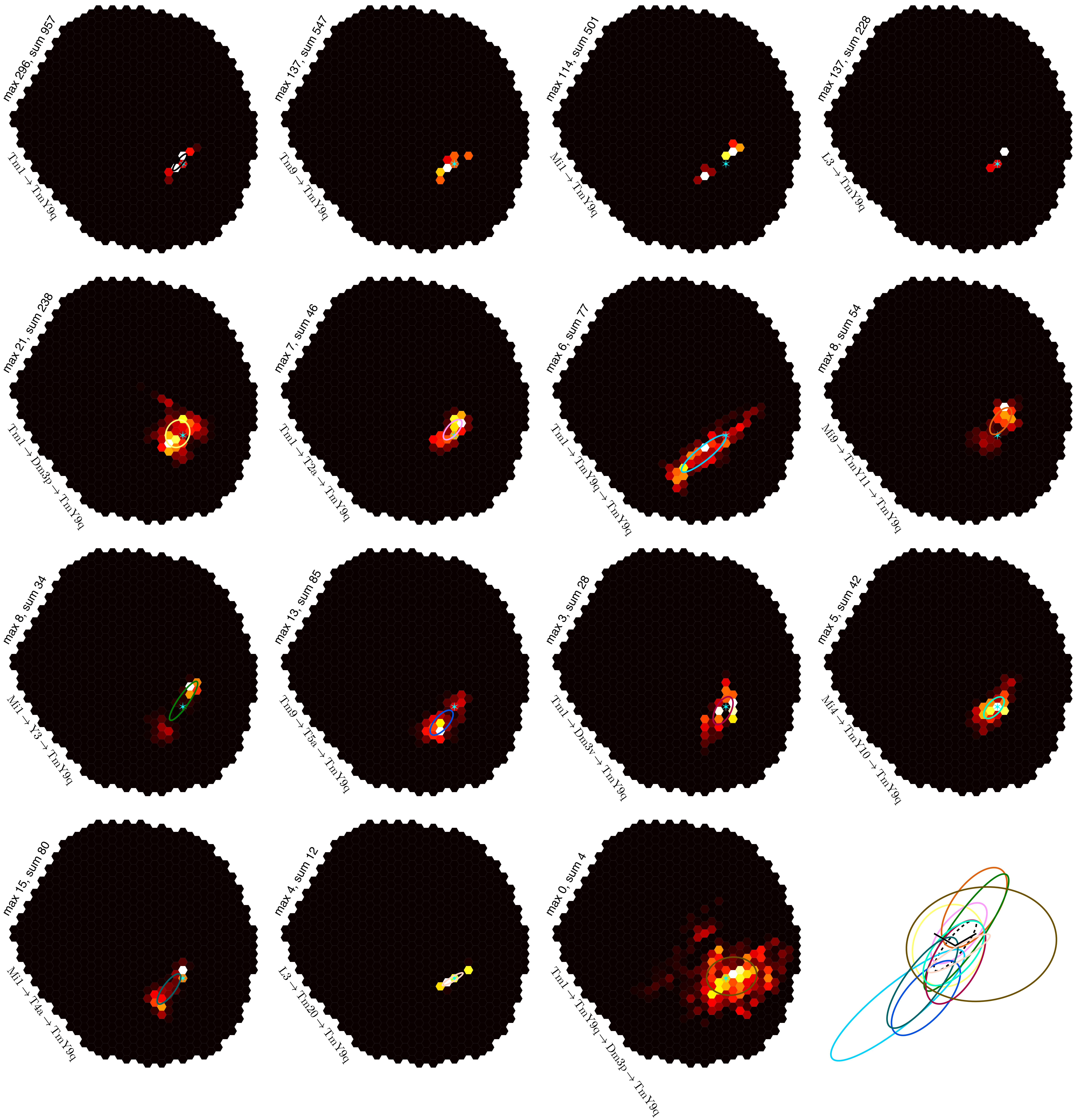
max 296, sum 957
Tm1 → TmY9q
max 137, sum 547
Tm9 → TmY9q
max 114, sum 501
Mi1 → TmY9q
max 137, sum 228
L3 → TmY9q
max 21, sum 238
Tm1 → Dm3p → TmY9q
max 7, sum 46
Tm1 → T2a → TmY9q
max 6, sum 77
Tm1 → TmY9q → TmY9q
max 8, sum 54
Mi9 → TmY11 → TmY9q
max 8, sum 34
Mi1 → Y3 → TmY9q
max 13, sum 85
Tm9 → T5a → TmY9q
max 3, sum 28
Tm1 → Dm3v → TmY9q
max 5, sum 42
Mi4 → TmY10 → TmY9q
max 15, sum 80
Mi1 → T4a → TmY9q
max 4, sum 12
L3 → Tm20 → TmY9q
max 0, sum 4
Tm1 → TmY9q → Dm3p → TmY9q
